# Spatiotemporal variation and decomposition of early neonatal mortality in Ethiopia using demographic health survey data

**DOI:** 10.1038/s41598-026-37784-5

**Published:** 2026-02-06

**Authors:** Habtamu Dessie Mitiku

**Affiliations:** Department of Statistics, College of Natural and Computational Sciences, Injibara University, Injibara, Ethiopia

**Keywords:** Early neonatal mortality, Spatio-temporal analysis, Decomposition analysis, Ethiopia, Diseases, Health care, Medical research, Risk factors

## Abstract

Early neonatal mortality is a serious public health issue in Ethiopia. Therefore, this study aimed to map the regional disparities and identify factors contributing to early neonatal mortality in Ethiopia over time. Ethiopian demographic and health survey (2000–2019) dataset were utilized. A total of 80,286 early neonates was included in this study. Logit based decomposition analysis was employed to understand the contributing factors for the change in early neonatal mortality over time. Getis Ord GI* statistic was performed to identify the cold and hot spots of the early neonatal mortality in Ethiopia. In addition, kriging interpolation was used to predict the burden of early neonatal mortality in the unsampled areas of the country based on the observed data. Early neonatal mortality trends in Ethiopia has been decline from 43 in 2000 to 33 in 2019 per 1000 live births. It was spatially clustered, with significant hotspots in the Benishangul Gumuz and some areas of Oromia, Tigray, Amhara and Somali regions. In the logit multivariable decomposition analysis babies born in rural (B − 0.0002, 95% CI − 0.002 to − 0.001), Women had partner (B − 0.0005, 95% CI − 0.0007 to − 0.0003), preceding birth interval ≥ 2 years (B − 0.008, 95% CI − 0.005 to − 0.001), Health facility delivery (B − 0.001, 95% CI − 0.003 to − 0.001), had ANC visits (B − 0.02, 95% CI − 0.03 to − 0.01), early initiations of breastfeeding (B − 0.002, 95% CI − 0.003 to − 0.001), multiple pregnancies (B − 0.002, 95% CI − 0.003 to − 0.001), and mothers education higher (B − 0.004, 95% CI − 0.006 to − 0.002) were a substantial factors that contribute to the change in the decline in early neonatal mortality in Ethiopia over time. Strengthen maternal and newborn care, expand skilled birth attendance, enhance antenatal and postnatal services, and utilize spatial evidence to inform targeted policy and resource allocation.

## Introduction

Early neonatal mortality (NMR) occurs when a newborn dies within the first seven days of life, and remains a significant global health challenge^1–3^. In 2022, World Health Organization (WHO) reported that approximately 2.3 million newborns died within the first 20 days of life, translating to nearly 6500 deaths daily^4^. This alarming rate contributes to about 47% of all child deaths under age five^4^. Notably, 75% of these neonatal deaths occur within the first week, with around 1 million newborns dying within the initial 24 h of life^4^. A systematic review study also shows that approximately 38.8% of neonatal death occurred within the first 24 h^5^ About 52.4%^6^ and 60%^7^ of neonatal deaths occur within the first 2 and 3 days of life respectively. Although substantial progress has been made since 1990, resulting the reduction of neonatal deaths from 5 million to 2.3 million by 2022^4^. The decline has been slower compared to post-neonatal under-5 mortality rates. Furthermore, the pace of improvement has slowed significantly since 2010. According to the WHO report 64 countries are projected to miss the Sustainable Development Goals (SDG) target for neonatal mortality by 2030, unless they take urgent interventions^4^.

In Sub-Saharan Africa, the risk of death in early neonatal mortality is 60 times (higher than lowest mortality countries^4^. In 2022, the WHO report shows that early neonatal mortality ranged from 0.7 death per 1000 live births to 39.4 deaths per 1000 live births. South Africa 21 deaths per 1000 live births^8^, Egypt 14 deaths per 1000 live births^9^, Rwanda19 deaths per 1000 live births^10^, Kenya 21 deaths per 1000 live births^11^, Ghana 23 deaths per 1000 live births^12^, Uganda 25 deaths per 1000 live births^13^ and Tanzania 24 deaths per 1000 live births^14^.

Despite Ethiopia implementing various programs and policies to reduce neonatal mortality, there is a need for continuous efforts to improve healthcare access and quality for newborns in the country. According to the Ethiopia Demographic and Health Survey (EDHS) 2016 report the national early neonatal mortality was 41.8 deaths per 1000 live births^2^. Achieving the national target, as set by the Health Sector Transformation Plan II, to reduce neonatal mortality to 21 deaths per 1000 live births by 2025 is challenging^15^.

Research findings show that prematurity^16–18^, not initiation of breastfeeding^1,19^, low birth weight^2,17^, birth asphyxia^5,16,18^, congenital anomalies^20–23^, infections^2,3,5,6,17,18,24^, Preceding birth interval^25^ and inadequate access to healthcare services^5,26^ are associated factors of early neonatal mortality. Furthermore, studies emphasize that strengthening healthcare systems includes investments in infrastructure, healthcare workforce training, access to essential medicines and equipment, and community-based healthcare initiatives to improve early neonatal outcomes^27–29^.

The early neonatal mortality rate is an important indicator of healthcare quality and access. However, in Ethiopia, there is still a lack of comprehensive evidence on this issue and its determinants in different contexts. Therefore, this study aims to meticulously map the spatial and time variations and factors that contributing to the change in the decline in early neonatal mortality in Ethiopia to address this pressing concern.

## Methods

### Study setting, design, and period

The study focused on Ethiopia. The country has nine regions (Tigray, Afar, Amhara, Oromia, Somali, Benishangul-Gumuz, Southern Nations, Nationalities, and People’s Region (SNNP), Gambelia, Harari) and two city administrations.

The study utilized data from five consecutive nationally representative cross-sectional surveys (EDHS 2000–2019)^30–34^. The surveys used a two-stage stratified cluster sampling method, with the primary sampling units being clusters and households as the secondary sampling units.

### Source population

This study considered newborns aged 0 to 7 days of childbirth as the source population. In this study, data on newborns were extracted from the birth record (BR file) dataset, which included 29,217 in 2000, 26,364 in 2005, 28,823 in 2011, 26,646 in 2016, and 9236 in 2019.

### Study variables

#### Outcome variable

The outcome variable for this study was early neonatal death status, which was dichotomized, as ‘yes’ = 1 for newborns who died within seven days of life and ‘no’ = 0 for newborns who were alive within seven days of life.

#### Independent variables

Factors include region, residence (recoded as urban, and rural), religion (recoded as Orthodox, Muslim, and Others), marital status (recoded as had no partner, had partner), maternal age (recoded as < 20, 20–34, 35–49), maternal education (recoded as no education, primary education, secondary, and Higher), sex of child (recoded as male, female), child live with whom (recoded as mother’s, others), place of delivery (recoded as home, health facility), the child is twin (recoded as single birth, Multiple births), When child put to the breast (recoded as immediately, not immediately), preceding birth interval (recoded as < 2 years, ≥ 2 years), antenatal care (ANC) visits during pregnancy (recoded as No, Yes), delivery by Caesarian section (No, Yes), size of child at birth (recoded as small, Average, Large) and as a grouping variable all the five year survey period was used.

### Data collection procedure

In this study, the data was obtained from the official measure DHS website (https://dhsprogram.com) after obtaining permission via an online request by specifying the analysis objective. The outcome and independent variables were extracted from the Birth Recode (BR) data set. The location data (latitude and longitude) was also accessed from the measure DHS program, with the coordinates displaced for confidentiality purposes.

### Data management and analysis

The data was cleaned and analyzed using STATA software (V.17). Summary statistics were generated to describe the study population. A logit-based multivariate decomposition analysis was employed to identify the factors contributing to the change in early neonatal mortality over the survey years. This analysis broke down the decrease in early neonatal mortality observed between surveys into a characteristics (or endowments) component and a coefficient (or effects of characteristics) component. The analysis was fitted in five phases (2000–2005, 2005–2011, 2011–2016, 2016–2019, and 2005–2019) using the Stata command (mvdcmp). In the bivariable logit decomposition analysis, variables with a *p* value of less than 20% were included in the multivariable logit decomposition analysis. Similarly, variables with a *p* value < 5% in the multivariate logit decomposition analysis were considered significant contributing factors for the decrease in early neonatal mortality over time.

ArcGIS version 10.8 software was used to explore the spatiotemporal pattern of early neonatal mortality in Ethiopia. The global spatial autocorrelation (Global Moran’s I) was used to assess whether early neonatal mortality was dispersed (− 1), clustered (+ 1), or randomly (close to 0) distributed in the study area. A statistically significant Moran’s I (*p* < 0.05) showed that early neonatal mortality is non-random. Hot spot analysis (Getis Ord GI* statistic) was also performed to identify the cold and hot spots of early neonatal mortality. In addition, the Kriging interpolation method was used to predict the burden of early neonatal mortality in the unsampled areas of the country based on the observed data.

## Results

### Socio-demographic and obstetric related characteristics of early neonates in Ethiopia

In the five consecutive surveys, more than 98% of mothers gave birth single. Nearly 90% of babies were born at home, and majority (87%) were born to mothers living in rural areas. The proportion of mothers who had antenatal car visits increased from 31.93% in 2000 to 74.56% in 2019. Besides, a significant number (85%) of mothers had no education and a small proportion (1.31%) of mothers had higher education. Regarding the preceding birth interval, almost (67%) of mothers gave birth with more than two years space (Table 1).

**Table 1:** Socio-demographic and obstetric related characteristics of early neonates in Ethiopia EDHS (2000–2019).

Characteristics	EDHS years				
	2000	2005	2011	2016	2019
	Frequency (%)	Frequency (%)	Frequency (%)	Frequency (%)	Frequency (%)
Region					
Tigray	3179 (10.88)	2796 (10.67)	3298 (11.10)	2794 (10.49)	756 (8.19)
Afar	1607 (5.50)	1571 (5.96)	2930 (9.86)	2487 (9.33)	1038 (11.24)
Amhara	4755 (16.27)	4172 (15.82)	3789 (12.75)	2896 (10.87)	892 (9.66)
Oromia	5839 (19.98)	5260 (19.95)	4458 (15.00)	4192 (15.73)	1235 (13.37)
Somali	1912 (6.54)	1747 (6.76)	2656 (8.94)	3583 (13.45)	1165 (12.61)
Benenshangul Gumz	2173 (7.44)	1782 (6.76)	2593 (8.73)	2317 (8.70)	884 (9.57)
SNNP	4468 (15.29)	4981 (18.89)	4281 (14.41)	3434 (12.89)	1065 (11.53)
Gambelia	1484 (5.08)	1237 (4.69)	2029 (6.83)	1626 (6.10)	636 (6.89)
Harari	1405 (4.81)	1146 (4.35)	1449 (4.88)	1338 (5.02)	665 (7.20)
Addis Ababa	1092 (3.74)	700 (2.66)	672 (2.26)	723 (2.71)	298 (3.23)
Dire Dawa	1303 (4.46)	972 (3.69)	1563 (5.26)	1256 (4.71)	602 (6.52)
Residence					
Urban	3741 (12.80)	2867 (10.87)	4055 (13.64)	3741 (14.04)	1689 (18.29)
Rural	25,476 (87.20)	23,497 (89.13)	25,663 (86.36)	22,905 (85.96)	7547 (81.71)
Mother’s Age in years					
< 20	491 (1.68)	515 (1.95)	513 (1.73)	384 (1.44)	228 (2.47)
20–34	15,255 (52.21)	14,199 (53.86)	16,615 (55.91)	15,238 (57.19)	6174 (66.85)
35–49	13,471 (46.11)	11,650 (44.19)	12,590 (42.36)	11,024 (41.37)	2834 (30.68)
Religion					
Orthodox	12,703 (43.48)	10,434 (39.58)	9470 (31.87)	7911 (29.69)	2466 (26.70)
Muslim	11,525 (39.45)	10,279 (38.99)	13,864 (46.65)	13,428 (50.39)	4971 (53.82)
Others	4989 (17.08)	5651 (21.43)	6384 (21.48)	5307 (19.92)	1799 (19.48)
Marital status					
Had not a partner	2268 (7.76)	1542 (5.85)	2086 (7.02)	1413 (5.30)	324 (3.51)
Had partner	26,949 (92.24)	24,822 (94.15)	27,632 (92.98)	25,233 (94.70)	8912 (96.49)
Maternal highest educational level					
No education	25,086 (85.86)	21,614 (81.98)	22,595 (76.03)	19,676 (73.84)	6054 (65.55)
Primary	2865 (9.81)	3504 (13.29)	6263 (21.07)	5331 (20.01)	2397 (25.95)
Secondary	1193 (4.08)	1089 (4.13)	593 (2.00)	1112 (4.17)	498 (5.39)
Higher	73 (0.25)	157 (0.60)	267 (0.93)	527 (1.98)	287 (3.11)
When child put to breast					
Immediately	14,651 (50.15)	18,946 (71.86)	16,612 (55.90)	19,348 (72.61)	6894 (74.64)
Not-immediately	14,566 (49.85)	7418 (28.14)	13,106 (44.10)	7298 (27.39)	2342 (25.36)
Child is twin					
Single	28,639 (98.02)	25,918 (98.31)	28,977 (97.51)	26,093 (97.92)	9035 (97.82)
Multiple	578 (1.98)	446 (1.69)	741 (2.49)	553 (2.08)	201 (2.18)
Sex of child					
Male	15,086 (51.63)	13,716 (52.03)	15,249 (51.31)	13,727 (51.52)	4676 (50.63)
Female	14,131 (48.37)	12,648 (47.97)	14,469 (48.69)	12,919 (48.48)	4560 (49.37)
Child lives with whom					
Mother	20,649 (88.18)	20,411 (90.80)	23,035 (89.74)	21,476 (89.40)	8057 (93.63)
Others	2768 (11.82)	2068 (9.20)	2634 (10.26)	2547 (10.60)	548 (6.37)
Preceding birth interval in years					
< 2years	6451 (29.16)	6104 (30.60)	7094 (31.91)	6636 (33.52)	2187 (32.75)
≥ 2years	15,669 (70.84)	13,842 (69.40)	15,137 (68.09)	13,164 (66.48)	4490 (67.25)
Had antenatal care visits during pregnancy					
No	4742 (67.32)	4323 (67.62)	4060 (54.85)	2298 (34.20)	638 (25.44)
Yes	2249 (31.93)	2070 (32.38)	3342 (45.15)	4422 (65.80)	1870 (74.56)
Place of delivery					
Home	9564 (90.59)	8588 (89.97)	9607 (86.67)	6830 (68.56)	2074 (52.06)
Health facility	994 (9.41)	957 (10.03)	1478 (13.33)	3132 (31.44)	1910 (47.94)
Delivery by caesarean section					
No	10,359 (98.85)	9388 (98.36)	10,813 (97.55)	9681 (97.18)	3752 (94.18)
Yes	120 (1.15)	157 (1.64)	272 (2.45)	281 (2.82)	232 (5.82)
Child size at birth					
Small	3575 (33.87)	2652 (27.78)	3469 (31.29)	2737 (27.47)	Na
Average	3695 (35.00)	3920 (41.07)	4369 (39.41)	4200 (42.16)	Na
Large	3286 (31.13)	2973 (31.15)	3247 (29.29)	3025 (30.37)	Na

Na: data not available.

### Trends of early neonatal mortality in Ethiopia

**Fig. 1:**
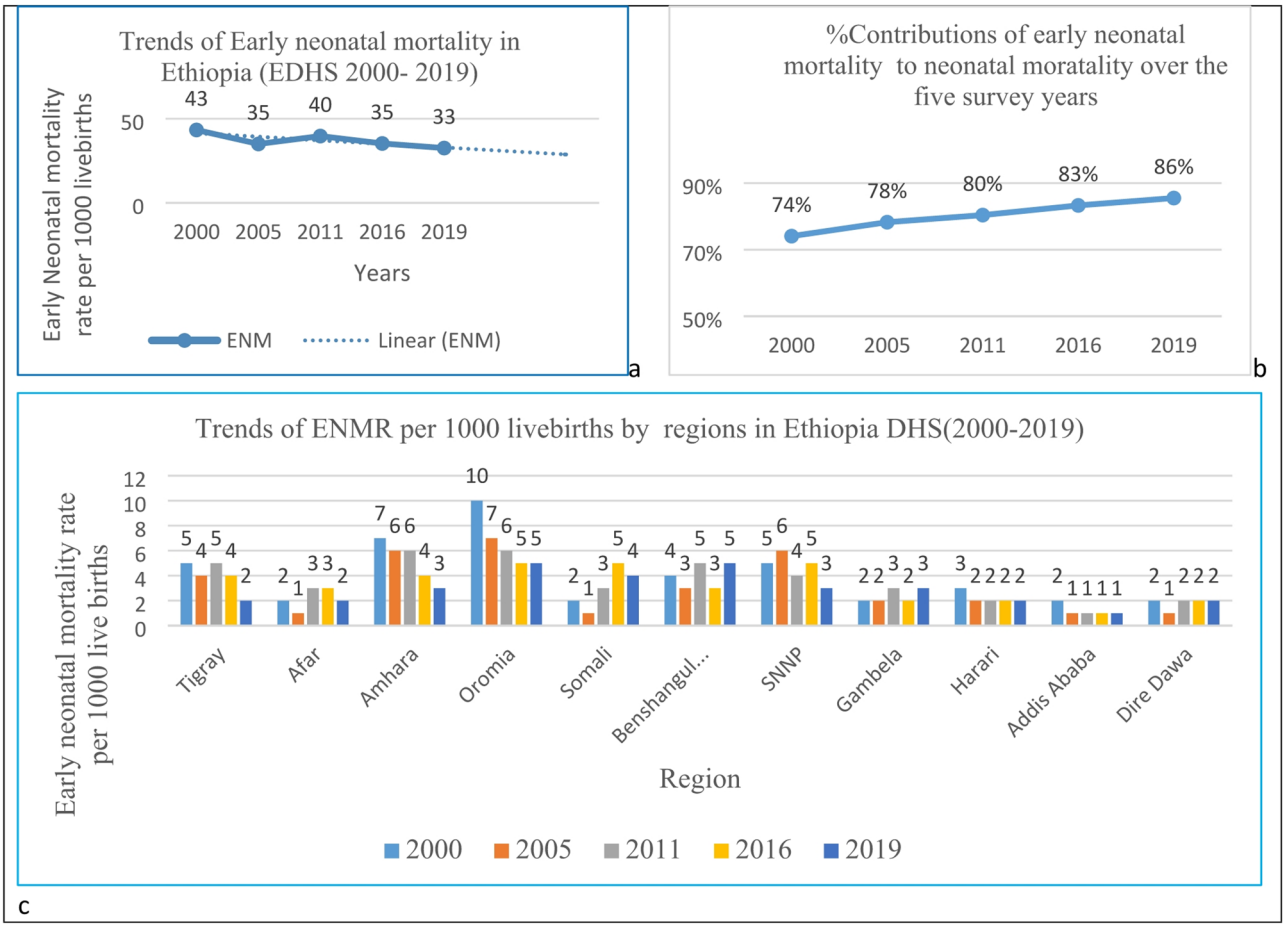
Trends of early neonatal mortality in Ethiopia EDHS 2000–2019.

The temporal trends of early neonatal mortality rate in Ethiopia over the study period (2000–2019) showed a significant decline, which is decreased from 43 per 1000 live births in 2000 to 33 per 1000 live births in 2019. The decline was observed in the survey period 2000–2005 with a 8 per 1000 live births drop down and in the survey period, 2011–2019 decreased from 40 to 33 per 1000 live births, which is a 82.5% decline, and it remains almost stagnant in the last two surveys (Fig. 1a).

Despite the decline in early neonatal mortality over the survey period, the percentage contributions of early neonatal mortality to neonatal mortality shown increased over the five years (74%, 78%, 80%, 83% and 86%) respectively (Fig. 1b). Also, Benshangul-Gumze and Oromia had shown higher proportion of early neonatal mortality disparities (Fig. 1c).

### Percentage point differences in early neonatal mortality across contexts in Ethiopia

The rate of decline in early neonatal mortality from 2000 to 2019 varied in terms of different factors. For example, the reduction in the stated period was the highest (2.05%) in the Tigray regional state and the lowest (0.42%) in the Afar Regional State of Ethiopia. Despite the decline, there is an increment in early neonatal mortality (− 0.67%) in the Somali region of Ethiopia. Besides, the decline was higher (0.03%) in urban and lower (1.31%) in rural settlements. The coverage of antenatal care visit was increased from 31.9 to 74.6% in the last decade, which had an important compositional contribution to the decline of early neonatal mortality by 0.21% (Table 2).

**Table 2:** Percentage point difference in early neonatal mortality in Ethiopia from EDHS (2000–2019).

Variables	2000 n = 29,217	2005 n = 26,364	2011 n = 28,823	2016 n = 26,646	2019 n = 9236	Percentage point difference in early neonatal mortality				
						(a)	(b)	(c)	(d)	(e)
Region										
Tigray	3.9	3.61	4.29	2.61	1.85	− 0.29	0.68	− 1.68	− 0.76	− 2.05
Afar	2.92	2.04	3.06	3.06	2.5	− 0.88	1.02	0	− 0.56	− 0.42
Amhara	3.49	3	3.79	3.04	1.68	− 0.49	0.79	− 0.75	− 1.36	− 1.81
Oromia	4.66	3.25	3.32	2.89	3.72	− 1.41	0.07	− 0.43	0.83	− 0.94
Somali	2.25	1.66	2.91	3.21	2.92	− 0.59	1.25	0.3	− 0.29	0.67
Gambelia	3.37	3.39	4.42	2.34	2.36	0.02	1.03	− 2.08	0.02	− 1.01
SNNP	3.07	3.00	2.43	2.97	1.78	− 0.07	− 0.57	0.54	− 1.19	− 1.29
Benshangul Gumz	4.97	3.09	5.27	3.15	3.62	− 1.88	2.18	− 2.12	0.47	− 1.35
Harari	4.27	3.49	2.94	3.36	2.41	− 0.78	− 0.55	0.42	− 0.95	− 1.86
Addis Ababa	1.83	0.86	1.18	1.52	0.67	− 0.97	0.32	0.34	− 0.85	− 1.16
Dire Dawa	2.53	2.06	3.33	3.11	1.33	− 0.47	1.27	− 0.22	− 1.78	− 1.2
Maternal age										
< 20	2.05	1.55	1.55	1.82	1.75	− 0.5	0	0.27	− 0.07	− 0.3
20–34	3.67	2.77	2.77	2.85	2.46	− 0.9	0	0.08	− 0.39	− 1.21
35–49	3.65	3.17	3.17	3.08	2.54	− 0.48	0	− 0.09	− 0.54	− 1.11
Residence										
Urban	2.22	1.85	2.47	2	2.19	− 0.37	0.62	− 0.47	0.19	− 0.03
Rural	3.83	3.05	3.63	3.08	2.52	− 0.78	0.58	− 0.55	− 0.56	− 1.31
Marital status										
Had not a partner	3.54	2.85	2.85	2.26	0.93	− 0.5	0	0.27	− 0.07	− 0.3
Had partner	3.64	2.92	2.92	2.97	2.51	− 0.9	0	0.08	− 0.39	− 1.21
Maternal highest education										
No education	3.81	3.03	3.72	3.04	2.44	− 0.78	0.69	− 0.68	− 0.6	− 1.37
Primary	2.93	2.74	2.82	2.78	2.71	− 0.19	0.08	− 0.04	− 0.07	− 0.22
Secondary	1.68	1.84	1.52	2.61	1.81	0.16	− 0.32	1.09	− 0.8	0.13
Higher	1.37	0	2.23	0.95	0	− 1.37	2.23	− 1.28	− 0.95	− 1.37
Religion										
Orthodox	3.5	2.92	3.73	2.74	2.15	− 0.58	0.81	− 0.99	− 0.59	− 1.35
Muslim	3.8	2.87	3.37	3.13	2.67	− 0.93	0.5	− 0.24	− 0.46	− 1.13
Others	3.55	3.01	3.32	2.69	2.33	− 0.54	0.31	− 0.63	− 0.36	− 1.22
Child sex										
Male	4.21	3.57	4.05	3.64	3.12	− 0.64	0.48	− 0.41	− 0.52	− 1.09
Female	3.01	2.21	2.86	2.17	1.78	− 0.8	0.65	− 0.69	− 0.39	− 1.23
Child is twin										
Single	3.2	2.68	3.06	2.65	2.25	− 0.52	0.38	− 0.41	− 0.4	− 0.95
Multiple	24.57	17.04	19.71	16.09	11.94	− 7.53	2.67	− 3.62	− 4.15	− 12.63
Preceding birth interval										
< 2 years	5.60	4.78	4.78	4.19	3.57	− 0.82	0	− 0.59	− 0.62	− 2.03
≥ 2 years	2.26	1.67	1.16	1.65	1.42	− 0.59	− 0.51	0.49	− 0.23	− 0.84
When child put to breast										
Immediately	3.81	2.76	3.41	2.77	2.55	− 1.05	0.65	− 0.64	− 0.22	− 1.26
Not-immediately	3.44	3.34	3.56	3.34	2.18	− 0.1	0.22	− 0.22	− 1.16	− 1.26
Had antenatal care visits during pregnancy										
No	99.54	99.31	99.31	99.04	99.53	− 0.23	0.00	− 0.27	0.49	− 0.01
Yes	0.22	0.24	0.24	0.45	0.01	0.02	0.00	0.21	− 0.44	− 0.21
Place of delivery										
Home	2.13	1.48	1.95	1.77	1.78	− 0.65	0.47	− 0.18	0.01	− 0.35
Health facility	1.41	1.04	1.41	1.6	1.2	− 0.37	0.37	0.19	− 0.4	− 0.21
Delivery by caesarian section										
No	2.07	1.44	1.89	1.72	1.55	− 0.63	0.45	− 0.17	− 0.17	− 0.52
Yes	2.5	1.27	1.45	1.42	0.86	− 1.23	0.18	− 0.03	− 0.56	− 1.64
Size of child at birth										
Smaller	2.01	1.17	2.01	2.01	Na	− 0.84	0.84	0.00	Na	Na
Average	1.6	1.33	1.31	1.88	Na	− 0.27	− 0.02	0.57	Na	Na
Larger	2.65	1.82	2.48	1.72	Na	− 0.83	0.66	− 0.76	Na	Na
Prevalence	3.63	2.92	3.47	2.93	2.46	− 1.29	0.55	− 1.46	− 1.53	− 1.83
95% CI	3.32, 3.85	2.72, 3.13	3.27, 3.69	2.73, 3.14	2.15, 2.79	− 1.40, − 1.28	0.55, 0.56	− 1.46, − 1.45	− 1.42, − 1.65	− 1.93, − 2.90

(a): phase I (2005–2000), (b): phase II (2011–2005), (c): phase III (2016–2011), (d): phase IV (2019–2016), (e): phase V (2019–2000), Na: data not available.

### Decomposition analysis

The multivariate logit decomposition analysis revealed that 58.2% of the decrease in early neonatal mortality was due to the difference in coefficient (difference in the effect of characteristics), while 41.8% of decrease was due to the difference in composition of the respondent (endowment) across the surveys (Table 3). Among the change due to composition (endowment); change in the composition of babies born to mothers lived in rural (B = − 0.0002, 95% CI − 0.002, − 0.001), to those born to mothers had partners (B = − 0.0005, 95% CI − 0.0007, − 0.0003), babies born to mothers with primary, secondary and higher education (B = − 0.004, 95% CI − 0.006, − 0.002), babies from mother had ≥ 2 years of preceding birth interval (B = − 0.008, 95% CI − 0.005, − 0.001), babies not immediately put breast (B = − 0.002, 95% CI  − 0.003, − 0.001), delivery at health facility (B =  − 0.001, 95% CI  − 0.003, − 0.001), had ANC follow up during pregnancy (B =  − 0.02, 95% CI − 0.03, − 0.01), and multiple pregnancies (B =  − 0.002, 95% CI − 0.003, − 0.001), were significantly contributed for the decrease in early neonatal mortality over 19 years (from 2000 to 2019). Among the decrease in early neonatal mortality attributed to the difference in coefficients; the difference in effects of those babies born to mothers who had higher education (B = − 0.002, 95% CI − 0.003, − 0.001), babies from mothers had ≥ 2 years of preceding birth interval (B = − 0.002, 95% CI − 0.003, − 0.001), multiple pregnancies (B = − 0.001, 95% CI − 0.003, − 0.001), and babies born to mother had ANC visits during pregnancy (B = 0.02, 95% CI 0.001, 0.04) were the factors significantly contributed for the decrease in early neonatal mortality in Ethiopia. Keeping all other variables constant, the improvement of women’s educational status to primary and above before the survey had a positive significant contribution to the decline of the trend. Likewise, babies born to women who had ≥ 2 years preceding birth interval had a positive impact to decline early neonates’ death and single pregnancies had a significant contribution to the decline of early neonatal death. Early neonates born to mother living in rural areas had a high risk of early neonatal death (Table 4).

**Table 3:** The overall decomposition analysis result of the decrease in early neonatal mortality over the last 19 years in Ethiopia EDHS (2000–2019).

Early neonatal mortality	Coefficient (95% CI)	Pct
E	− 0.003 (− 0.005, − 0.001)	41.1
C	− 0.02 (− 0.03,− 0.01)	58.9
R	− 0.03 (− 0.04, − 0.02)	

C-coefficient, CI-confidence interval, E-Endowment, Pct.-percentage contribution, R-Residual.

**Table 4:** Multivariate logistic regression decomposition analysis of early neonatal mortality in Ethiopia EDHS (2000–2019).

Variables	Difference due to characteristics (E)		Difference due to coefficient (C)	
	Coeff (95% CI)	Pct	Coeff (95% CI)	Pct
Maternal age				
< 20years	1		1	
20–34	0.0001 (− 0.0001, 0.0003)	− 0.01	− 0.004 (− 0.02, 0.01)	0.4
35–49	0.006 (− 0.001, 0.01)	− 0.02	− 0.003 (− 0.02, 0.02)	0.3
Residence				
Urban	1		1	
Rural	− 0.0002 (− 0.002, − 0.001)*	7.4	− 0.01 (− 0.02, 0.01)	− 0.0001
Marital status				
Had no partner	1		1	
Had partner	− 0.0005 (− 0.0007, − 0.0003)*	1.4	0.02 (− 0.01, 0.05)	0.6
Maternal highest education				
No education	1		1	
Primary	− 0.001 (− 0.005, − 0.003)*	− 0.7	0.001 (− 0.0004, 0.002)	0.9
Secondary	− 0.0002 (− 0.002, − 0.001)*	1.4	0.001 (− 0.0004, 0.002)	0.9
Higher	− 0.004 (− 0.006,− 0.002)*	1.7	− 0.002 (− 0.003, − 0.001)*	12.6
Child sex				
Male	1		1	
Female	0.003 (− 0.002, 0.008)	− 0.01	0.007 (− 0.006, 0.02)	0.5
Preceding birth interval in years				
< 2years	1		1	
≥ 2years	− 0.008 (− 0.005,− 0.001)**	3.2	− 0.002 (− 0.003,− 0.001)*	10.4
When child put to breast (early initiation of breast feeding)				
Immediately	1		1	
Not-immediately	− 0.002 (− 0.003, − 0.001)*	1.4	− 0.01 (− 0.02, 0.01)	− 0.001
Had antenatal visits during pregnancy				
No	1		1	
Yes	− 0.02 (− 0.03,—0.01)**	10.4	0.02 (0.01, 0.04)*	19.2
Place of delivery				
Home	1		1	
Health facility	− 0.001 (− 0.003, − 0.001)**	11.8	0.001 (− 0.006, 0.006)	0.9
Delivery by caesarian section				
No	1		1	
Yes	0.001 (− 0.09, 0.093)	− 0.01	− 0.0002 (− 0.0007, 0.0003)	0.4
Child is twin				
Single	1		1	
Multiple	− 0.002 (− 0.003, − 0.001)**	3.2	− 0.001 (− 0.003, − 0.001)*	11.8
Overall		41.1		58.9

*Is if *p* value < 0.05 and ** if *p* value < 0.01.

### Spatio-temporal patterns of early neonatal mortality in Ethiopia EDHS (2000–2019)

**Fig. 2:**
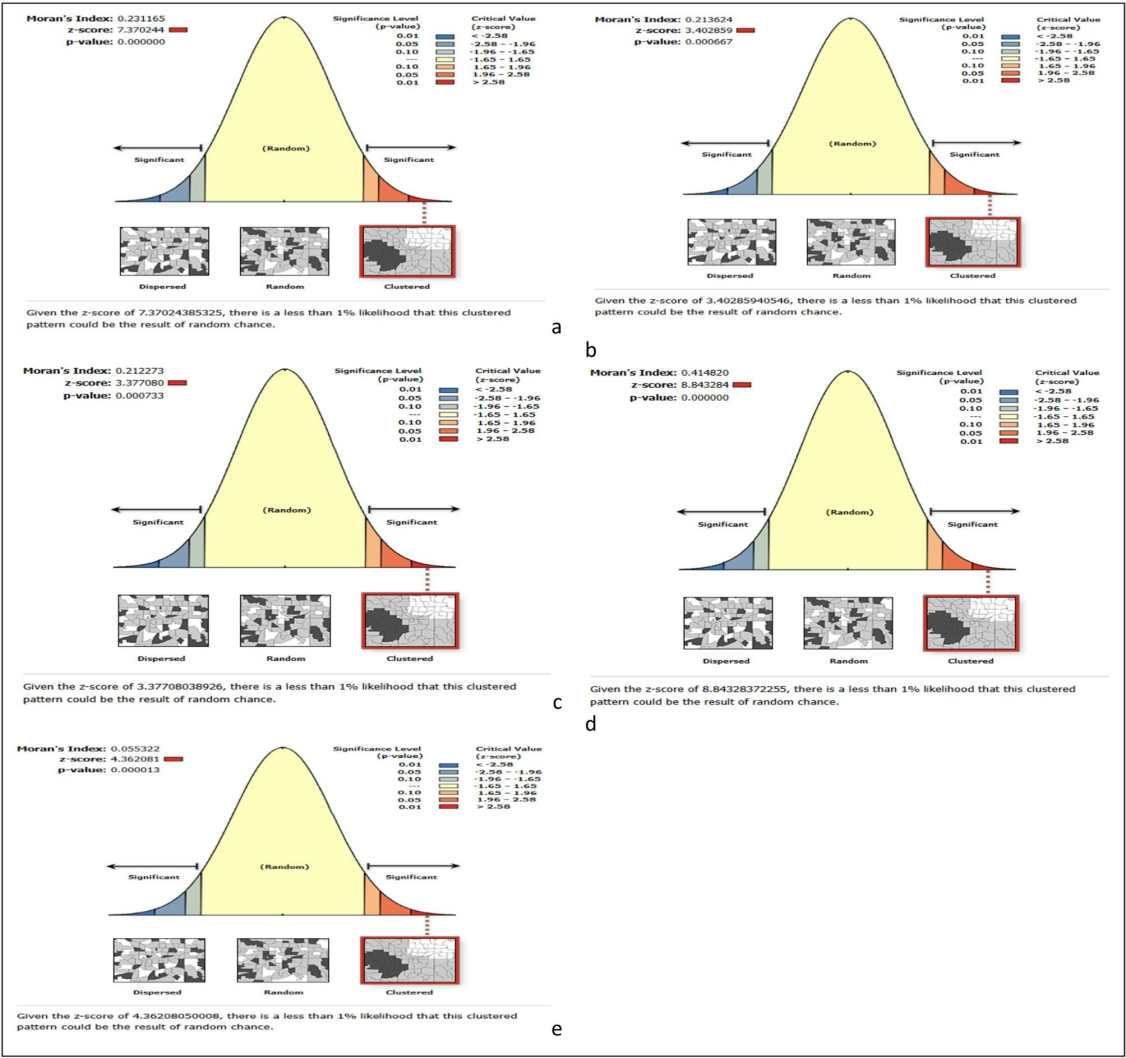
Spatial autocorrelation of early neonatal mortality in Ethiopia: (**a**) 2000EDHS, (**b**) 2005EDHS, (**c**) 2011EDHS, (**d**) 2016EDHS, (**e**) 2019EDHS.

The spatial distribution of early neonatal mortality in Ethiopia was non-random among the five consecutive surveys. The global Moran’s I value was 0.23 (*p* value < 0.001) in 2000, 0.21 (*p* value < 0.001) in 2005, 0.52 (*p* value < 0.001) in 2011, 0.41 (*p* value < 0.001) in 2016, and 0.10 (*p* value < 0.001) in 2019 Ethiopian Mini Demographic and health surveys. The global spatial autocorrelation revealed a clustering pattern of early neonatal mortality across the EAs (Moran’s index = 0.192459, z-score = 4.289037, *p* value = 0.000018) (Fig. 2a–e).

### The Hot spot analysis of early neonatal mortality in Ethiopia over the five surveys

**Fig. 3:**
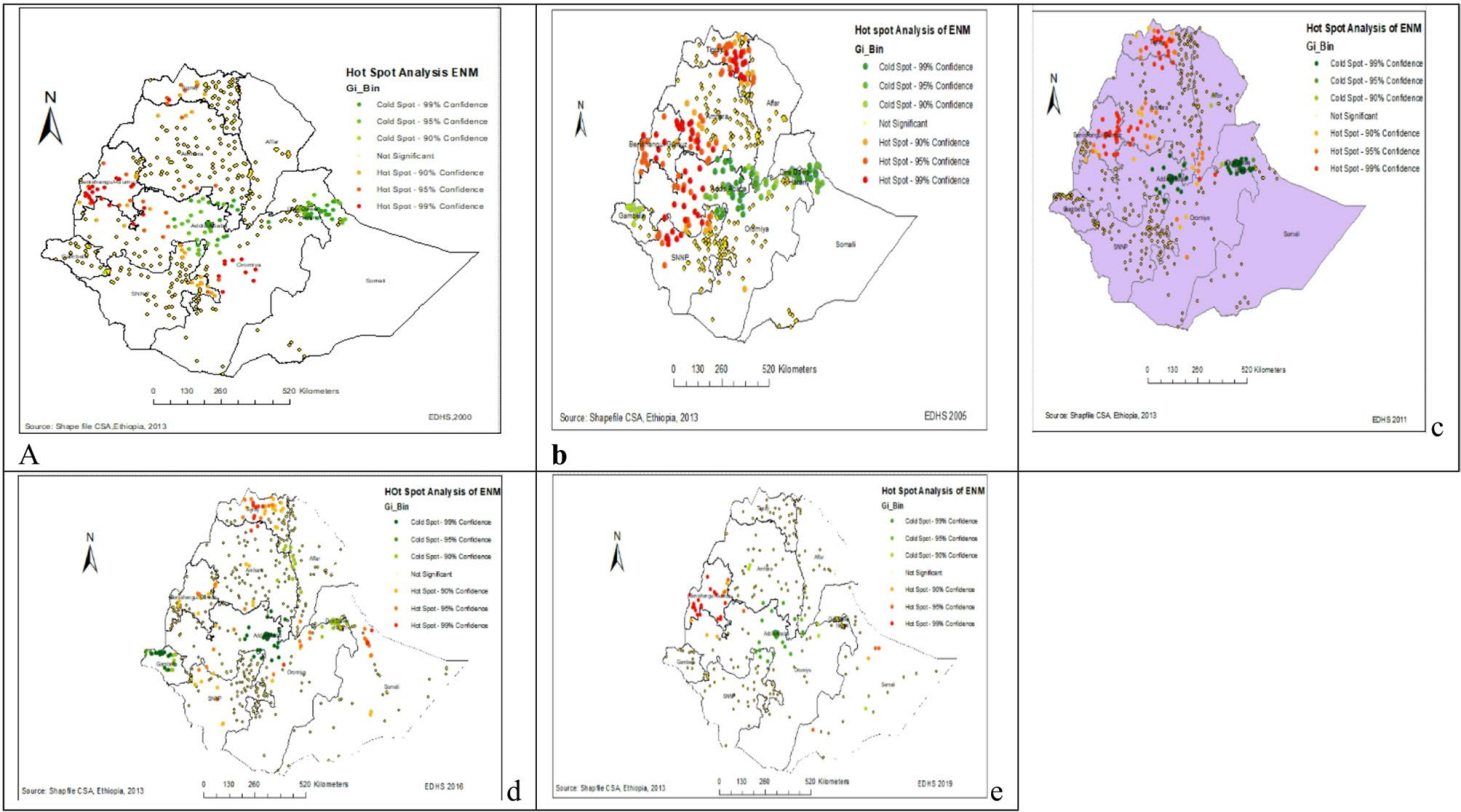
Hot Spot analysis for early neonatal mortality in Ethiopia: (**a**) 2000EDHS, (**b**) 2005EDHS, (**c**) 2011EDHS, (**d**) 2016EDHS, (**e**) 2019EDHS.

The spatial distribution of early neonatal mortality in Ethiopia was different in the five survey years. In EDHS 2000, a high proportion of early neonatal mortality was detected mainly at Benishangul Gumz, Oromia, Amhara and Tigray regional states of Ethiopia. In EDHS 2005, a high proportion of early neonatal mortality was detected mainly at Benishangul Gumz, Tigray Amhara, Oromia and SNNPR regional states of Ethiopia. In EDHS 2011, high clustering of early neonatal mortality detected in most parts of Tigray, Amhara, Afar, Benishangul and SNNPR, and western part of the Oromia region of Ethiopia. Likewise, in EMDHS 2019, a high proportion of early neonatal mortality detected Benishangul Gumz, and Somali region of Ethiopia (Fig. 3a–e).

### Predicting early neonatal mortality for unsampled areas of Ethiopia (2000–2019)

**Fig. 4:**
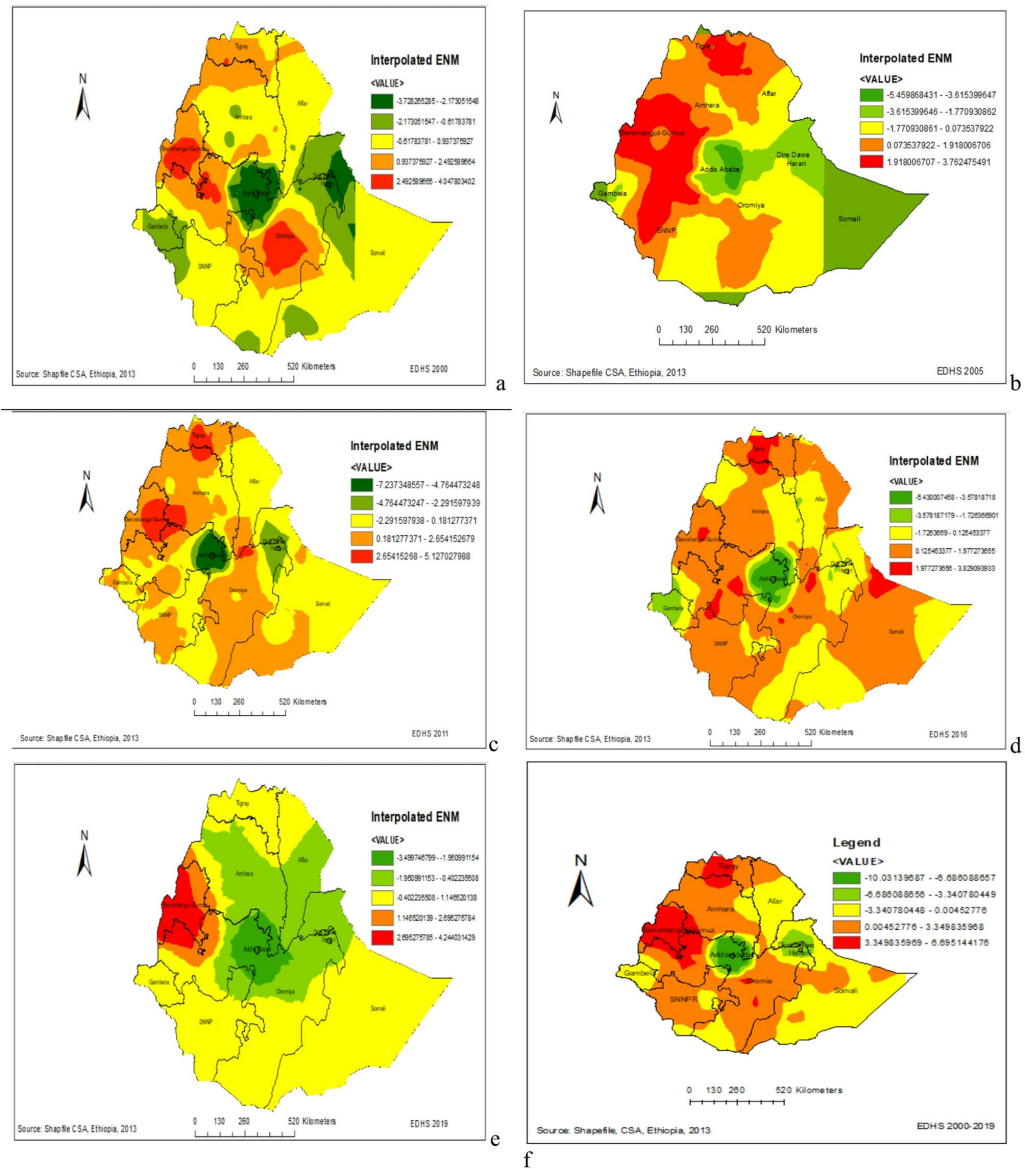
The Kriging interpolation based predicted geospatial map for early neonatal mortality in Ethiopia: (**a**) 2000EDHS, (**b**) 2005EDHS, (**c**) 2011EDHS, (**d**) 2016EDHS, (**e**) 2019EDHS, (**f**) Combined 2000–2019EDHS.

The kriging interpolation analysis predicted that early neonatal mortality was relatively higher in northwestern, central, and southeast Benshangul Gumz. Similarly, southwest and northeast parts of the Oromia region also had high mortality rates. In 2005 and 2011 EDHS, Benshangul Gumz and Tigray region had a relatively higher early neonatal mortality rate. In the EDHS 2016 survey, Tigray, some parts of Benshangul Gumz, Oromia, and Gambelia region had relatively higher early neonatal mortality rates. On the other hand, Addis Ababa and Dire Dawa have lower early neonatal mortality in Ethiopia (Fig. 4a–f).

## Discussion

Early neonatal mortality is a significant contributor to childhood mortality in Ethiopia. The spatiotemporal analysis shows that early neonatal mortality rates vary across different regions of Ethiopia. The kriging interpolation results indicate that Benshanbul Gomez had a higher proportion of early neonatal mortality. Over the past nineteen years, there has been a 76.7% decrease in early neonatal mortality, from 43 per 1000 live births in 2000 to 33 per 1000 live births in 2019. Among the declines in early neonatal mortality, 58.2% were attributable to the difference in coefficients, while 41.1% of the decrements were attributed to women’s compositional characteristics. However, the percentage contribution of early neonatal mortality to the overall neonatal mortality increased from 74% in 2000 to 86% in 2019.

The prevalence of early neonatal mortality in this study was higher compared to other African countries such as Egypt^9^, Kenya^11^, and Nigeria^29^, which reported 14, 22, and 32 early neonatal deaths per 1000 live births, respectively. This difference could be attributed to variations in healthcare systems across the countries. In comparison to Ethiopia, Kenya and Nigeria have higher rates of hospital beds and physicians per 1000 inhabitants^2^.

Following multivariable logit decomposition analysis, among the changes due to composition (endowment): babies born in rural, those born to mothers who had partners, babies born to mothers who had education, babies from mothers had ≥ 2 years of preceding birth interval, babies not immediately put breastfeed, delivery at a health facility, had ANC follow up, and multiple pregnancies were significantly contributed for the decrease in early neonatal mortality over 19 years (from 2000 to 2019). Likewise, babies born to mothers who had higher education, babies from mothers who had ≥ 2 years of preceding birth interval, multiple pregnancies, and babies born to mothers who had ANC visits during pregnancy were among the changes in coefficients that significantly contributed to the decrease in early neonatal mortality in Ethiopia.

In this study, early neonatal mortality was more predominant in rural than urban areas of the country^5,19,24–26^. This difference could be attributed to variations in transportation, access to information about perinatal health service utilization, and health service availability in rural.

In this study, the log odds of early neonatal mortality were higher among babies born to mothers who had no partner compared with babies born to mothers who had a partner. Previous studies also had similar findings^26^. This association could be linked to the issue of low maternal dietary diversity practice during pregnancy in the risk of low birth weight and preterm birth^2,17,35^.

Early neonatal mortality was more likely to happen among newborn babies born at home than babies born at a health facility. This study was consistent with previous studies^18,19,24,25^. This happens due to a lack of access to all components of essential newborn care and immediate postpartum care services at home. Therefore, in low-income countries like Ethiopia, promoting and ensuring facility-based childbirth will improve the survival of both babies and mothers^6,16,17,22,27,28^. In addition, giving at health facilities had an advantage in reducing a potential delay and enhancing early management of obstetric complications and birth asphyxia^5,18,22^. Furthermore, babies delivered at health facilities had a chance to initiate early breastfeeding. This ensures the survival of the babies^6,24,25^.

Regarding babies born with mothers who had no education were more likely to die in the first week of life compared with those born with women who had education^1,6,21,23^. These could be mothers’ understanding and knowledge of the access to healthcare-seeking behavior for neonatal health issues. The lack of child healthcare facilities and their poor quality may also increase the danger of death for low birthweight neonates^16,28^.

In this study, babies born to mothers who had antenatal care visits during pregnancies have more survival in the first seven days of life than babies born to mothers who had no antenatal care visit. The current finding agreed with previous studies^3–6,15–19,22–29^. This finding proved that ANC is a pivotal element in averting death. On the other hand, babies born to mothers who had a preceding birth interval of less than two years were more likely to increase the death of neonates^6^. This could be the mother not having enough time to fully recover from the previous pregnancy, resulting in a risk of preterm birth, low birth weight, anemia, and preeclampsia. Therefore, to improve the survival of early neonates, working on knowledge creation and educating mothers had a positive outcome in minimizing the risk.

The findings found that multiple pregnancies were significantly associated with early neonatal death. The occurrence of early neonatal death was higher among neonates born from multiple pregnancies compared to those born from singleton pregnancies. Babies from multiple pregnancies may experience growth restrictions, low Apgar scores, and extremely low birth weights^5^. Furthermore, multiple pregnancies are more likely to lead to complications during pregnancy, labor, and after birth^2^.

## Conclusion

Early neonatal mortality in Ethiopia declined slowly over the survey period, with significant spatiotemporal variations observed across regions. Thus, public health interventions and programs targeting the identified clusters, mothers’ education, rural babies, women who had less than two years preceding birth interval, multiple pregnancies, had no antenatal care visits, and home delivery would be helpful in maintaining the declining trend of early neonatal mortality.

## Data Availability

The dataset supporting the conclusions of this article is publicly accessible through the MEASURE DHS program, which can be accessed at http://www.dhsprogram.com. Upon clarification of the study’s objectives, authorization can be obtained, enabling free download of the data.

## Abbreviations

CI: Confidence interval
EA: Enumeration areas
EDHS: Ethiopian Demographic and Health Survey
ENMR: Early neonatal mortality rate
ANC: Antenatal care
SDG: Sustainable development goal
SNNP: Southern nations and nationalities of people
